# Salmon Calcitonin Exerts an Antidepressant Effect by Activating Amylin Receptors

**DOI:** 10.3389/fphar.2022.826055

**Published:** 2022-02-14

**Authors:** Jian Jiang, Jun Ju, Liang Luo, Ze Song, Huanquan Liao, Xiuyan Yang, Shoupeng Wei, Dilong Wang, Wenhui Zhu, Jinlong Chang, Junzhe Ma, Hao Hu, Jiezhong Yu, Huiqing Wang, Sheng-Tao Hou, Shupeng Li, Huiliang Li, Ningning Li

**Affiliations:** ^1^ Tomas Lindahl Nobel Laureate Laboratory, The Seventh Affiliated Hospital, Sun Yat-Sen University, Shenzhen, China; ^2^ Department of Critical Care Medicine, The Seventh Affiliated Hospital, Sun Yat-sen University, Shenzhen, China; ^3^ Oncology Department, The Seventh Affiliated Hospital, Sun Yat-sen University, Shenzhen, China; ^4^ The Clinical Neuroscience Center, The Seventh Affiliated Hospital, Sun Yat-sen University, Shenzhen, China; ^5^ China-UK Institute for Frontier Science, Shenzhen, China; ^6^ Wolfson Institute for Biomedical Research, Division of Medicine, Faculty of Medical Sciences, University College London, London, United Kingdom; ^7^ The Fourth People’s Hospital of Datong City, Datong, China; ^8^ The Fifth People’s Hospital of Datong City, Datong, China; ^9^ Brain Research Centre and Department of Biology, School of Life Sciences, Southern University of Science and Technology, Shenzhen, China; ^10^ State Key Laboratory of Oncogenomics, School of Chemical Biology and Biotechnology, Peking University Shenzhen Graduate School, Shenzhen, China

**Keywords:** behavior test, depression, salmon calcitonin, chronic restraint stress, amylin receptor, AC187

## Abstract

Depressive disorder is defined as a psychiatric disease characterized by the core symptoms of anhedonia and learned helplessness. Currently, the treatment of depression still calls for medications with high effectiveness, rapid action, and few side effects, although many drugs, including fluoxetine and ketamine, have been approved for clinical usage by the Food and Drug Administration (FDA). In this study, we focused on calcitonin as an amylin receptor polypeptide, of which the antidepressant effect has not been reported, even if calcitonin gene-related peptides have been previously demonstrated to improve depressive-like behaviors in rodents. Here, the antidepressant potential of salmon calcitonin (sCT) was first evaluated in a chronic restraint stress (CRS) mouse model of depression. We observed that the immobility duration in CRS mice was significantly increased during the tail suspension test and forced swimming test. Furthermore, a single administration of sCT was found to successfully rescue depressive-like behaviors in CRS mice. Lastly, AC187 as a potent amylin receptor antagonist was applied to investigate the roles of amylin receptors in depression. We found that AC187 significantly eliminated the antidepressant effects of sCT. Taken together, our data revealed that sCT could ameliorate a depressive-like phenotype probably *via* the amylin signaling pathway. sCT should be considered as a potential therapeutic candidate for depressive disorder in the future.

## Introduction

Major depressive disorder is a life-threatening chronic mental disease affecting more than 20% of the population worldwide (Yan et al., 2010). Depression is a psychiatric disorder characterized by low mood and lack of interest in work, life, and social activities, which often leads to suicidal attempts and suicides (Marinova et al., 2014). Unfortunately, the range of available medications for depression treatment in the clinic is relatively limited and traditionally restricted to fluoxetine and ketamine. However, fluoxetine works usually after a long treatment period of 2–4 weeks and is ineffective in patients with severe depression, *via* selectively inhibiting 5-hydroxytryptamine (5-HT) reuptake to enhance the extracellular 5-HT level (Hirschfeld, 2000; Al-Harbi, 2012). Ketamine exerts an antidepressant effect *via* different mechanisms, including but not limited to blocking *N*-methyl-d-aspartate (NMDA) receptor, activating α-amino-3-hydroxy-5-methyl-4-isoxazolepropionic acid (AMPA) receptor, and regulating synaptic plasticity (Newport et al., 2015; Zanos et al., 2018). Meanwhile, ketamine has certain severe side effects, e.g., hallucinogenic, addictive, and neurotoxic responses, preventing its widespread application in the clinic (Short et al., 2018). Therefore, there is an increasing demand to develop new medications with high antidepressant capacity and safety.

Recently, the potential of peptides, e.g., neuropeptide Y (NPY), vasopressin, and calcitonin-related peptides, to treat neuropsychiatric disorders has been reported. The latest study has shown that the mRNA level of NPY in the prefrontal cortex (PFC) and hippocampus regions was significantly decreased in the postmortem brain of depressed and suicide patients (Sharma et al., 2022). Moreover, another study reported that NPY exerts an antidepressant effect when administered intranasally in rats (Nahvi et al., 2021). Besides, vasopressin can be involved in the regulation of the hypothalamic–pituitary–adrenal (HPA) axis to play an antidepressant effect (Rana et al., 2021). Calcitonin gene-related peptide (CGRP) has proven efficacy for the preventive treatment of migraine that is associated with depression and has improved depressive-like behavior in mice with 15-day chronic restraint stress (CRS) by intracerebroventricular administration (Hashikawa-Hobara et al., 2015; Spierings et al., 2021) Among them, calcitonin and calcitonin-related peptides as small molecular peptides have been widely used in the treatment of neuropsychological diseases. Calcitonins, including calcitonin, CGRP, amylin, and adrenomedullin, are a class of peptide hormones generated in vertebrates. The coding sequences of calcitonin (32 amino acids) and CGRP (37 amino acids) are located in the same gene and formed into two mature peptides by alternative splicing (Amara et al., 1982; Rosenfeld et al., 1983). A previous report has indicated that CGRP treatment increased the swimming time in mice during a forced swimming test (FST) (Schorscher-Petcu et al., 2009). Concurrently, another study has demonstrated that an injection of CGRP prior to a period of 15-day chronic stress could reduce the immobility time, suggesting the antidepressant potential of CGRP (Hashikawa-Hobara et al., 2015). Furthermore, the antidepressant effects of CGRP disappeared after blocking the CGRP receptor (Hashikawa-Hobara et al., 2015). Conversely, the levels of CGRP were increased in the hippocampus, frontal cortex, and amygdala in the rat model of depression established by maternal deprivation (Angelucci et al., 2019). It is noted that in the clinical trial of depressive patients, calcitonin in the serum is reduced (Mathe et al., 2002). However, whether calcitonin could exert an antidepressant effect remains unclear.

Amylin receptors (AMYRs) consist of calcitonin receptor (CTR) dimerized with receptor activity-modifying proteins (RAMPs) (Hay et al., 2018). *Kalafateli* et al. reported that AMYRs are expressed in the whole brain including reward-processing brain areas (Kalafateli et al., 2021). AMYRs are involved in motivated ingestive behavior and alcohol drinking (Mietlicki-Baase et al., 2017; Kalafateli et al., 2021). Small-molecule AMYR agonists are considered effective treatment candidates, especially because they can handle crossing the blood–brain barrier and perform with high specificity (Sonne et al., 2021). Salmon calcitonin (sCT) is a therapeutic agent known for the treatment of osteoporosis and Paget’s disease. The efficacy and favorable safety have been verified after several decades of clinical practice (McLaughlin and Jialal, 2021). Recent studies have shown that sCT affects reward-related areas of the brain such as the laterodorsal tegmental area (LDTg), ventral tegmental area (VTA), and nucleus accumbens (NAc) shell to modulate alcohol-related behaviors in rodents (Zakariassen et al., 2020; Kalafateli et al., 2021). Furthermore, sCT can attenuate certain nicotine-induced behaviors by modulating AMYRs (Aranas et al., 2021). sCT is an agonist of AMYRs (i.e., AMYR polypeptide), and its role in depression remains to be elucidated.

In the present study, we aimed to verify the antidepressant potential of sCT. First, we evaluated the effectiveness of the mouse model with CRS, and we detected the level of calcitonin in the serum and cerebral cortex taken from the depressive-like mice. Second, we showed that the depressive phenotype was alleviated by an acute application of sCT. Finally, to validate the receptor-dependent antidepressant potential of sCT, we harnessed AC187, an antagonist of AMYR, to block the antidepressant effects of sCT.

## Methods and Materials

### Animals

Mice were housed in a pathogen-free SPFII animal facility in a condition-controlled room (23°C ± 1°C, 50% ± 10% humidity) at the Southern University of Science and Technology (SUSTech), Shenzhen, China. A 12-h light/dark cycle was automatically imposed. Mice were maintained in a group of six in each ventilated cage and given access to food and water *ad libitum*. All animal experiments were conducted according to the protocols approved by the Animal Care Committee at SUSTech. The ARRIVE (Animal Research: Reporting of *In Vivo* Experiments) guidelines were followed when animal experimentation was designed and performed. Male C57BL/6J mice were imported from Guangdong Laboratory Animal Center (Guangzhou, China).

### Chronic Restraint Stress Model

After being acclimated to the facility for a week, mice were subjected to CRS as previously described, with minor modifications (Seo et al., 2012). Briefly, mice were restrained in flexible cylindrical plastic tubes fitted to allow them to breathe. Going through CRS, mice were immobilized in the tube once for 2 h for 14 consecutive days. Mice assigned to a non-stressed group stayed in their own home cages.

### Open-Field Test

The open-field test (OFT) was used to measure the level of voluntary movement and anxiety (Choleris et al., 2001). Mice were put in the test room for 1 h to habituate to the environment. The open box (40 × 40 × 40 cm, L × W × H) was divided equally into 16 smaller grids, and the central four grids were set as the central area (20 × 20 cm). The distance that the mice traveled and the time that they spent in the central area were recorded for 10 min by EthoVision XT software (Noldus Information Technology, Leesburg, VA, USA).

### Elevated Plus Maze

The elevated plus maze (EPM) was formed with four arms, with open and closed arms crossed with each other, standing 1 m above the floor. The test mice were placed in the center facing the open arm, and their activity was measured for 5 min. The total time spent in open arms, center area, and closed arms was recorded with a video tracking system and analyzed by Noldus EthoVision XT10 software.

### Tail Suspension Test

Tail suspension test (TST) was performed according to the previously reported protocols (Ali et al., 2020; Li W et al., 2021). Briefly, mice were placed in the test room for 1 h to be familiarized with the environment. After that, the mouse tails were taped to the iron hook in the tail suspension box. EthoVision XT software was used to record the immobility time of mice within 6 min upon tail suspension.

### Forced Swimming Test

FST was performed according to previously reported protocols (Ali et al., 2020; Lee et al., 2021). Mice were placed in the test room for 1 h to be familiarized with the environment. The apparatus was a diaphanous cylindrical plexiglass container with a diameter of 11.5 cm and a height of 30 cm. Mice were individually placed in the water cylinder (water temperature: 22°C–24°C) for a 6 min test session, and the cumulative immobility time was counted and analyzed for the last 5 min. The immobility time was recorded by the EthoVision XT software. Mice were thought to be stationary when they floated motionless in the water or moved to keep their nose above the surface of the water.

### Three-Chamber Test

Social behaviors were adapted from experiments previously described (Sgritta et al., 2019). The three-chamber apparatus (60 × 40 × 20 cm, L × W × H) was divided into three interconnected chambers. Mice were first habituated for a 10-min period in three-chamber apparatus divided by transparent plexiglass. Sociability was evaluated during a second 10-min period in which the test mice could interact either with a small cage (Empty) in one chamber or a genotype, age, sex-matched stranger mouse (Mouse 1) that was placed in a cage on the other chamber. Subsequently, preference for social novelty was assayed, in a third 10-min period, by introducing a second stranger mouse (Mouse 2) into the previously empty cage. The time spent interacting with the empty cage or Mouse one or Mouse two was recorded and measured using the EthoVision XT 10 software package.

### Novel Object Recognition Test

The novel object recognition (NOR) task was adapted from experiments previously described (Leonzino et al., 2019). Mice were habituated to a box (40 × 40 × 40 cm, L × W × H) for 10 min and then returned to their home cage. Twenty-four hours after habituation, animals were exposed to two identical objects for 10 min exploration sessions in the same box. Two hours after object exploration, one object was replaced with a novel object, and the animals were then allowed to explore the two objects (i.e., novel vs. old) for 10 min. The time the mice spent sniffing within 2 cm of each object or directly touching the objects was recorded.

### Drug Administration

sCT (Tocris Bioscience, Bristol, United Kingdom) was diluted in 0.9% sterile saline and injected subcutaneously at 50 IU/kg (12.5 ug/kg) bodyweight 1 h before the behavioral test. Mice in the control group were injected with saline at the same volume. The AMYR antagonist AC187 (250 μg/kg, Cohesion Biosciences, London, United Kingdom) was injected intraperitoneally 10 min before the sCT administration. One hour after the sCT injection, the above behavioral tests were performed. The dosage and timing of the drug administration were determined based on previous studies (Kalafateli et al., 2019b).

### Mouse Calcitonin Measurement

Frozen cerebral cortex tissues were lysed with radioimmunoprecipitation assay (RIPA) buffer (Beyotime, Shanghai, China) and homogenized on ice. The supernatants were collected after centrifugation (Li W et al., 2021). mCT was measured by a sandwich ELISA (Jianglai Biological Technology Co., Ltd., Shanghai, China) in 100 μl of serum or supernatant of cortical homogenate according to the manufacturer’s protocols. Briefly, after the 96-well plate was cleaned 3 times using wash buffer, 100 µl of standard or sample was added to each well and incubated for 2 h at 37°C. Subsequently, the plate was washed 3 times using wash buffer and incubated using a biotin-conjugated antibody for 1 h at 37°C. Streptavidin–horseradish peroxidase (HRP) was added for a 30-min incubation at 37°C. Optical density was measured under 450 nm with an ELISA microplate reader.

### Western Blotting Assay

The brain tissues used to detect CTR expression were taken from the mice of the CRS and control groups that went through behavioral tests. Total protein was isolated from the dissected tissues using moderate-intensity RIPA buffer (Beyotime, Shanghai, China). After being centrifuged at 12,000 rpm at 4°C for 15 min, supernatants were collected, and protein concentration was determined using Pierce™ BCA Protein Assay Kit (Thermo Fisher Scientific, MA, United States). Proteins were separated by sodium dodecyl sulfate–polyacrylamide gel electrophoresis (SDS-PAGE), transferred to a polyvinylidene fluoride (PVDF) membrane (Merck Millipore, Guangzhou, China), and blocked in 5% fat-free milk in 1× TBST (0.1% Tween20 in Tris-buffered saline) for 1 h at room temperature. The blots were incubated with primary antibodies of CTR (Abcam, Cambridge, United Kingdom) in a 5% bovine serum albumin (BSA) solution overnight at 4°C. On the second day, the blots were washed with 1× TBST 3 times and incubated with HRP-conjugated secondary antibodies (anti-rabbit IgG, ProteinTech Group, Inc., Wuhan, China). Immunodetection was performed using a super ECL detection reagent (Yeasen Biotech, Shanghai, China), and the signal was detected with a ChemiDoc™ Touch Imaging System (Bio-Rad, Shanghai, China).

### Statistical Analysis

All data are represented as the mean ± SEM. Serous and cerebral cortical mCT levels and behavioral tests between the wild-type (WT) and CRS groups were analyzed by independent-samples *t*-test. The data of behavioral tests, among three groups, were analyzed with one-way ANOVA followed by Tukey’s multiple-comparisons test. Two-way ANOVA followed by Bonferroni’s multiple-comparisons test was used to analyze the results of the three-chamber social test and NOR test. Statistical analysis was performed using GraphPad Prism 8 (GraphPad Software, La Jolla, CA, United States), and *p* < 0.05 was considered statistically significant. The sample size calculation in this study was based on “AEEC animal experimentation sample size calculator” (http://www.lasec.cuhk.edu.hk/sample-size-calculation.html).

## Results

### Mice With CRS Showed Significant Depressive-Like Behaviors

**FIGURE 1:**
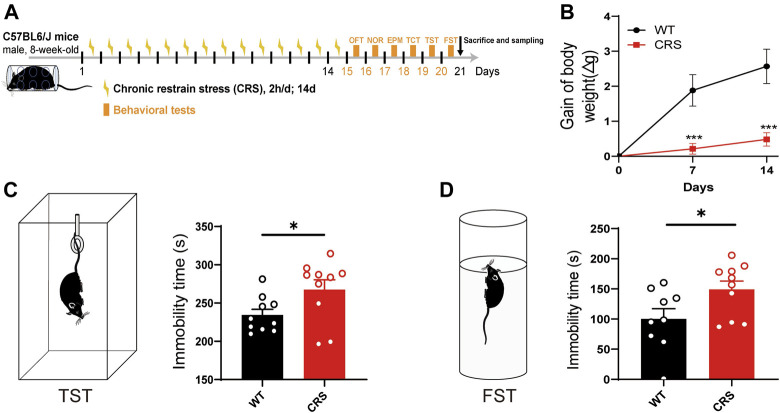
CRS mice showed a depressive-like phenotype. Schematic diagram showing experimental procedures of CRS mouse model **(A)**. Compared with WT controls, the weight of mice relative decreased after 7 or 14 days of CRS (WT: *n* = 10; CRS: *n* = 10) **(B)**. In the tail suspension experiment, the immobility time of CRS mice was significantly increased (WT: *n* = 10; CRS: *n* = 10) **(C)**. In FST, the immobility time of CRS mice was also increased (WT: *n* = 9; CRS: *n* = 10) **(D)**. The data were analyzed by unpaired *t*-test. *p < 0.05, ***p < 0.001. CRS, chronic restraint stress; WT, wild type; FST, forced swimming test.

To explore the therapeutic effects on depression, we established an animal model of CRS, which is widely used to screen for antidepressants (Campos et al., 2013; Li Y et al., 2021; Lee et al., 2021). In the present study, we treated male C57BL/6J mice with CRS for 2 weeks starting at the age of 8 weeks (Figure 1A). Compared to the control group, the body weight of mice was relatively reduced after 7 and 14 days of CRS [t_(18)_ = 4.072, *p* < 0.001; t_(18)_ = 4.462, *p* < 0.001, Figure 1B)], and thus the CRS model was primarily established, as the weight loss is often as a key indicator of depression. TST and FST were then performed in mice after 14 days of CRS. We found that the immobility time of CRS mice was significantly increased in both TST and FST [t_(18)_ = 2.268, *p* < 0.05, Figure 1C; t_(17)_ = 2.266, *p* < 0.05, Figure 1D], suggesting that our model of depression was successfully established. In addition, no significant deficits in social ability [F_(1, 36)_ = 0.740, *p* > 0.05, Supplementary Figure S1B] and cognitive memory [F_(1, 36)_ = 0.036, *p* > 0.05, Supplementary Figure S1E] were observed in this mouse model of depression.

### Locomotion and Anxiety Level did not Change in CRS Mice

**FIGURE 2:**
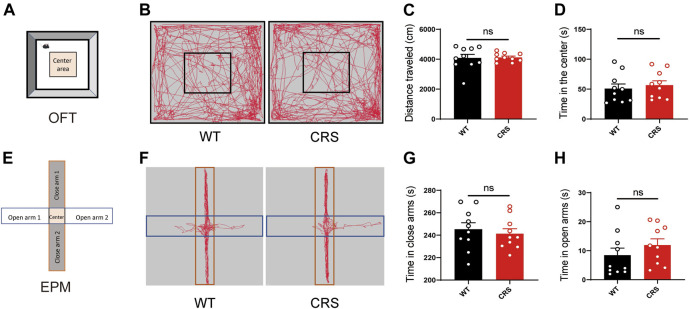
Locomotion and anxiety did not change in CRS mice. Scheme of the OFT **(A)**. Representative images of movement trace in OFT **(B)**. The distance traveled in OFT did not change in CRS mice (WT: *n* = 10; CRS: *n* = 10) **(C)**. Time in the center did not change compared with WT controls **(D)**. Scheme of the EPM **(E)**. Representative images of movement trace in EPM **(F)**. Time in closed arms did not change in CRS mice (WT: *n* = 10; CRS: *n* = 10) **(G)**. Time in the open arms did not change compared with WT controls **(H)**. The data were analyzed by unpaired *t*-test. CRS, chronic restraint stress; OFT, open field test; WT, wild-type; EPM, elevated plus maze.

Depressive and anxiety disorders are often associated, but the relationship between them has been controversial. In TST and FST, the immobility time may be highly linked to the ability of spontaneous movement in mice. To demonstrate that the increased immobility time of the CRS mice was attributed to depression but not to the effect of locomotion, we conducted the OFT to monitor the locomotion in the mice. We showed that there was no significant change in the distance traveled [t_(18)_ = 0.143, *p* > 0.05, Figures 2A–C]. The time that the CRS mice spent in the middle area, usually reflecting anxiety, did not alter either [t_(18)_ = 0.097, *p* > 0.05, Figure 2D]. In addition, we examined whether the CRS mice were accompanied by anxiety in the EPM. Of interest, there was no profound change in the time spent in the open and closed arms between the WT mice and the CRS mice [t_(18)_ = 0.550, *p* > 0.05, Figures 2E–G; t_(18)_ = 1.089, *p* > 0.05, Figure 2H].

### The Levels of Serous and Cortical Calcitonin were Decreased in CRS Mice

**FIGURE 3:**
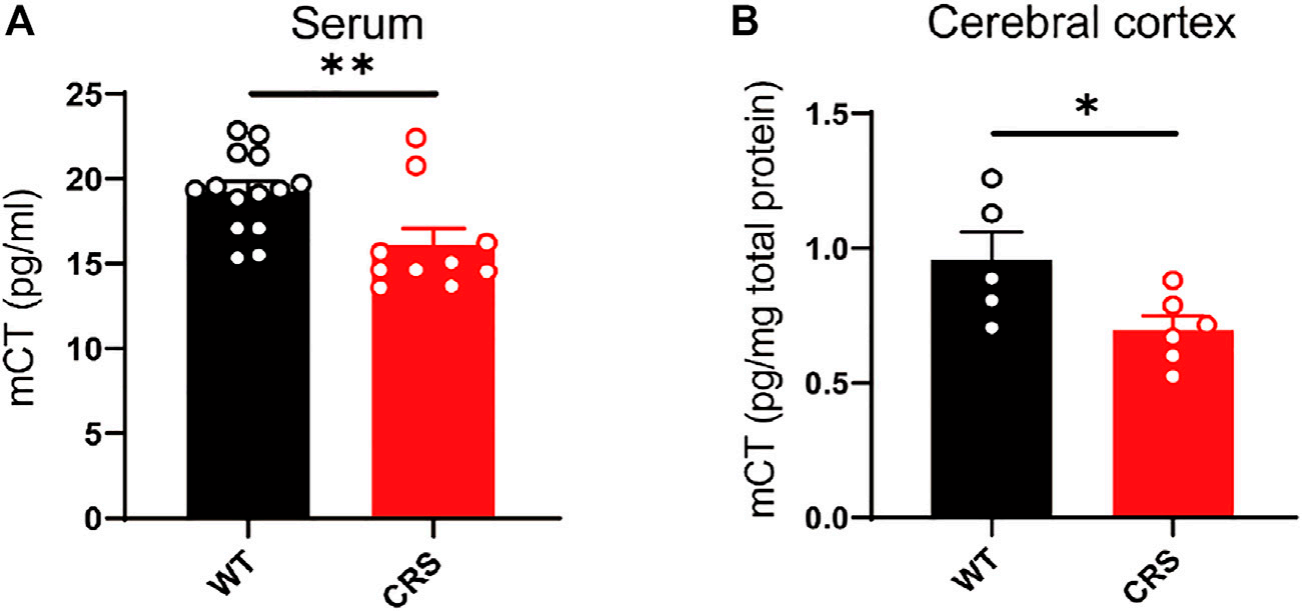
The level of mCT was reduced in the serum and cerebral cortex of CRS mice. mCT was reduced in the serum of CRS model (WT: *n* = 14; CRS: *n* = 10) **(A)**. mCT was reduced in the cerebral cortex of CRS model (WT: *n* = 5; CRS: *n* = 6) **(B)**. The data were analyzed by unpaired *t*-test. *p < 0.05, **p < 0.01. CRS, chronic restraint stress; WT, wild type.

In the clinical trial of depressive patients, calcitonin in the serum is reduced (Mathe et al., 2002). Here, we measured the mouse calcitonin (mCT) level in the serum and found its decline in the CRS mice compared to the control group [t_(22)_ = 2.832, *p* < 0.01, Figure 3A]. As the calcitonin is mainly produced in the parathyroid gland and could cross the blood–brain barrier, we also assessed the mCT level and found a decline in the cerebral cortex of the CRS mice [t_(9)_ = 2.39, *p* < 0.05, Figure 3B].

### Salmon Calcitonin did not Affect Locomotion and Anxiety of Wild-Type and CRS Mice

**FIGURE 4:**
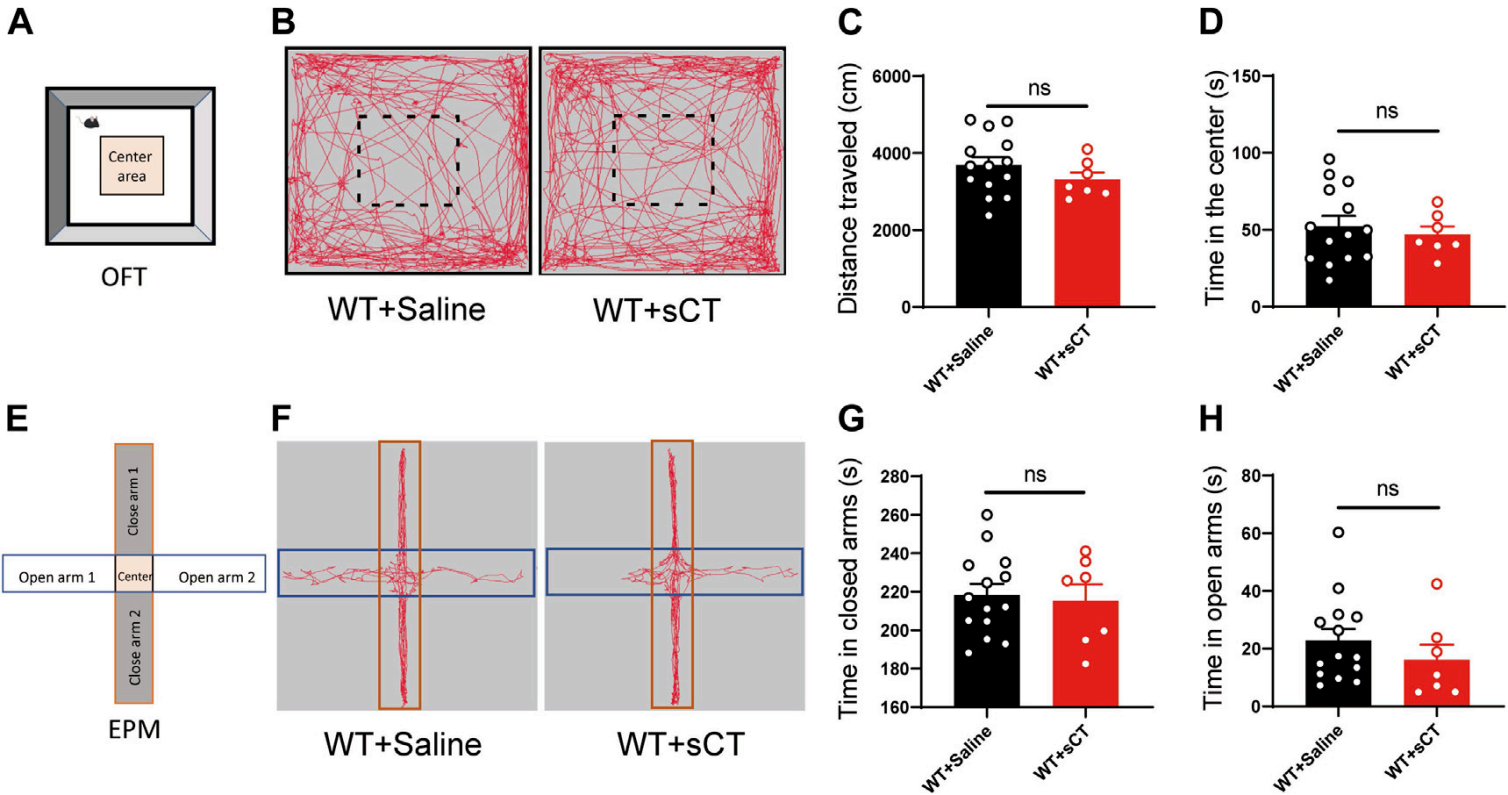
Salmon calcitonin (sCT) did not affect locomotion and anxiety level in WT mice. Scheme of the OFT **(A)**. Representative images of movement trace in OFT **(B)**. The distance travelled in OFT did not change with application of sCT (WT + saline: *n* = 14; WT + sCT: *n* = 7) **(C)**. Time in the center did not change with the application of sCT **(D)**. Scheme of the EPM **(E)**. Representative images of movement trace in EPM **(F)**. Time in closed arms did not change with application of sCT (WT + saline: *n* = 14; WT + sCT: *n* = 7) **(G)**. Time in open arms did not change with the application of sCT **(H)**. The data were analyzed by unpaired *t*-test. WT, wild type; OFT, open field test; EPM, elevated plus maze.

To exclude the possibility of sCT interference on the mouse locomotion, we injected sCT into the WT mice (i.e., non-stressed control) 1 h before the OFT to investigate whether it could affect spontaneous locomotion. We found that the distance that the WT mice traveled after the administration of sCT was not changed as compared to the control group injected with saline [t_(19)_ = 1.162, *p* > 0.05, Figures 4A–C]. At the same time, the time of WT mice into the center was not altered either [t_(19)_ = 0.541, *p* > 0.05, Figure 4D]. On the next day, we performed an EPM test on the same groups of mice 1 h after subcutaneous injection of sCT or saline. In the EPM test, the time that the mice stayed in the open arms or closed arms was not changed [t_(19)_ = 0.302, *p* > 0.05, Figures 4E–G; t_(19)_ = 0.984, *p* > 0.05, Figure 4H]. Similarly, we found that sCT did not affect locomotion and anxiety levels in CRS mice (Supplementary Figure S2).

### sCT Exerts Antidepressant Effects in CRS Mice

**FIGURE 5:**
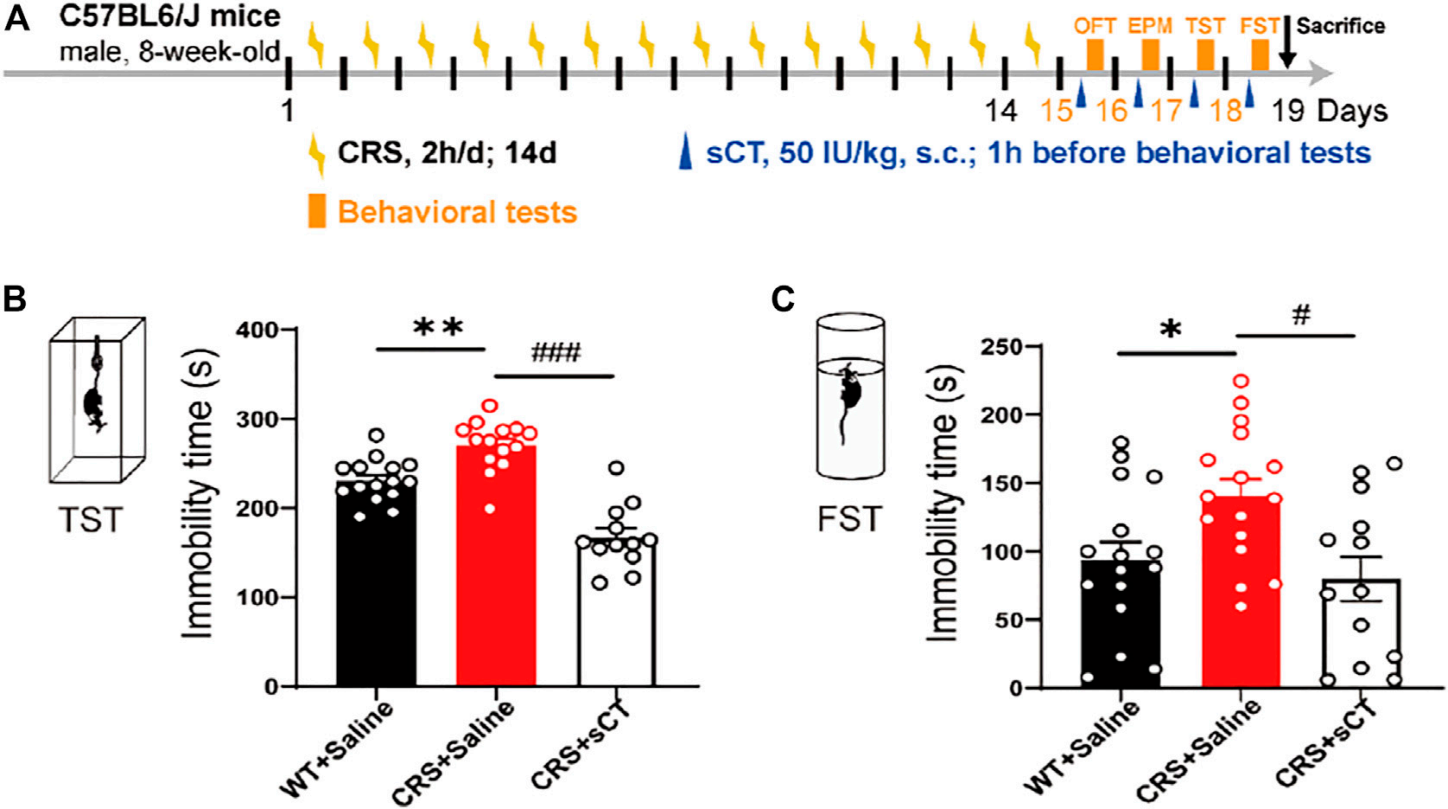
sCT exerted an antidepressant effect in CRS mice. Schematic illustration of the experimental design **(A)**. In TST, the immobility time of CRS mice was increased, while sCT could decrease the immobility time of CRS mice (WT + saline: *n* = 15; CRS + saline: *n* = 14; CRS + sCT: *n* = 12) **(B)**. In FST, the immobility time of CRS mice was increased, while sCT shortened immobility time in CRS mice (WT + saline: *n* = 16; CRS + saline: *n* = 16; CRS + sCT: *n* = 13) **(C)**. The data were analyzed by one-way ANOVA with Tukey’s multiple-comparisons test. *p < 0.05, **p < 0.01 vs. WT + saline; ^*#*^ *p* < 0.05, ^*###*^ *p* < 0.001 vs. CRS + saline. sCT, salmon calcitonin; CRS, chronic restraint stress; TST, tail suspension test; FST, forced swimming test.

Previous studies have recorded that the elimination half-life of sCT could persist for about 1–1.5 h (Chin et al., 2004; Bhandari et al., 2015). Thus, we applied a subcutaneous administration of sCT and did the behavior test 1 h afterward. Figure 5A shows the schematic illustration of the experimental design. The antidepressant effects of sCT were indicated by the responses in TST and FST. In the TST, the immobility time of the depressive-like mice was profoundly increased compared to that of the WT mice, while sCT administration significantly reduced such immobility time [F_(2, 38)_ = 0.324, *p* < 0.001, Figure 5B]. Similarly, in the FST, the immobility time of the CRS mice was increased, while the sCT administration rescued it significantly [F_(2, 44)_ = 0.444, *p* < 0.01, Figure 5C]. These results suggested that the sCT has antidepressant effects in CRS mice, while not affecting the locomotion and anxiety levels.

### Expression of CTR was not Altered in the Prefrontal Cortex, Cerebral Cortex (Without PFC), and Hippocampus of CRS Mice

**FIGURE 6:**
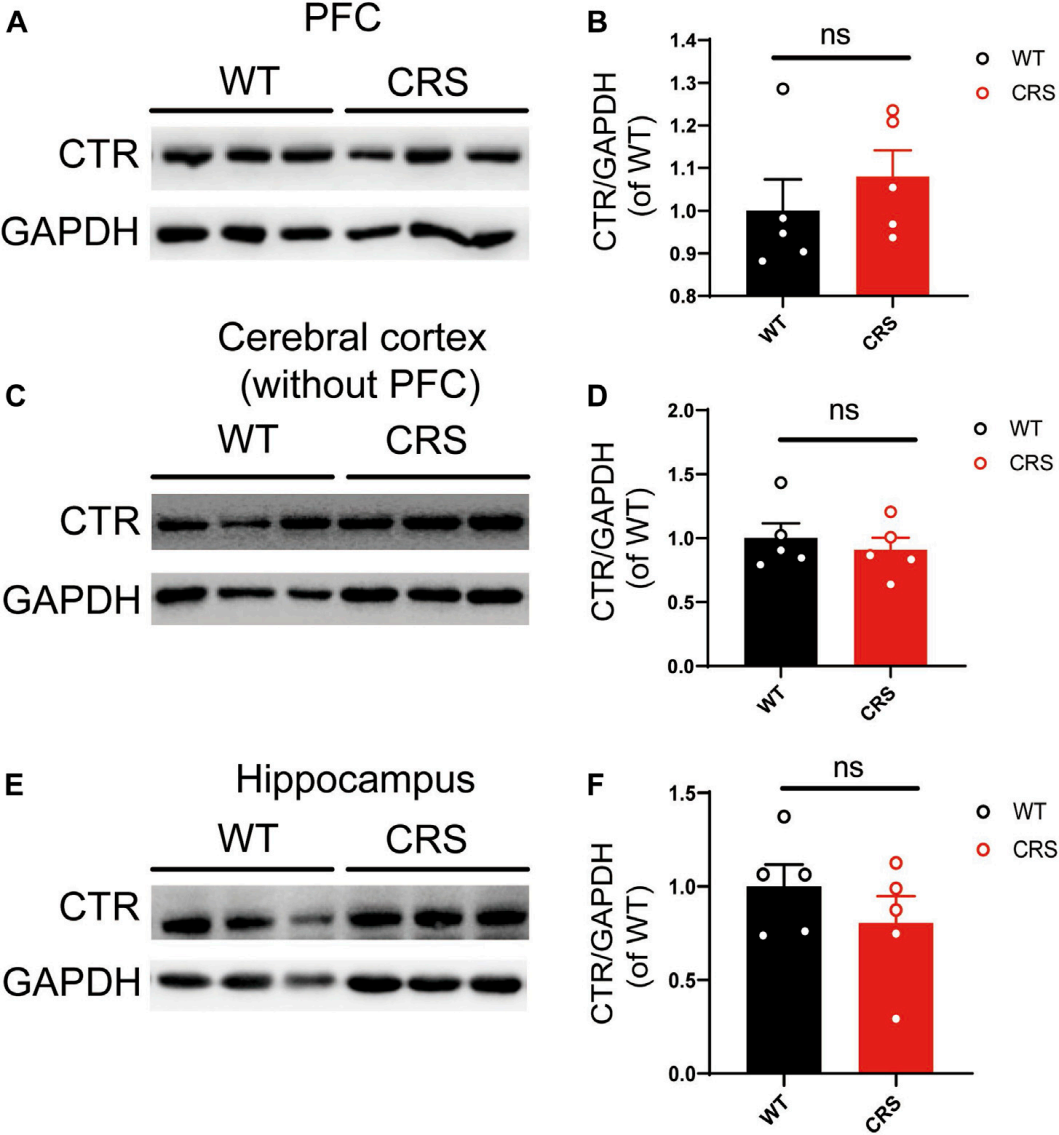
The level of CTR was not altered in PFC, cerebral cortex (without PFC), and hippocampus. The Western blotting bands in PFC **(A)**. Relative CTR expression was not altered in PFC (WT: *n* = 5; CRS: *n* = 5) **(B)**. The Western blotting bands in the cerebral cortex (without PFC) **(C)**. Relative CTR expression was not altered in the cerebral cortex (without PFC) **(D)**. The Western blotting bands in the hippocampus **(E)**. Relative CTR expression was not altered in the hippocampus **(F)**. The data were analyzed by unpaired *t*-test. CTR, calcitonin receptor; PFC, prefrontal cortex; WT, wild type; CRS, chronic restraint stress.

Considering the antidepressant effects of sCT, we sought to investigate if the expression of AMYR plays a role in the CRS condition. Hence, we performed Western blotting of CTR, one core component of AMYR, in PFC, cerebral cortex (without PFC), and hippocampus of the CRS mice vs. the WT controls. The expression of CTR was detected in all these three regions, and CTR protein levels were normalized to those of GAPDH. As illustrated in Figure 6, the CTR protein levels were not significantly changed in the PFC [t_(8)_ = 0.842, *p* > 0.05], cerebral cortex (without PFC) [t_(8)_ = 0.601, *p* > 0.05], and hippocampus [t_(8)_ = 1.052, *p* > 0.05] of CRS mice compared with unstressed mice.

### sCT Exerts Antidepressant Effects in the CRS Model by Activating the Amylin Receptor

**FIGURE 7:**
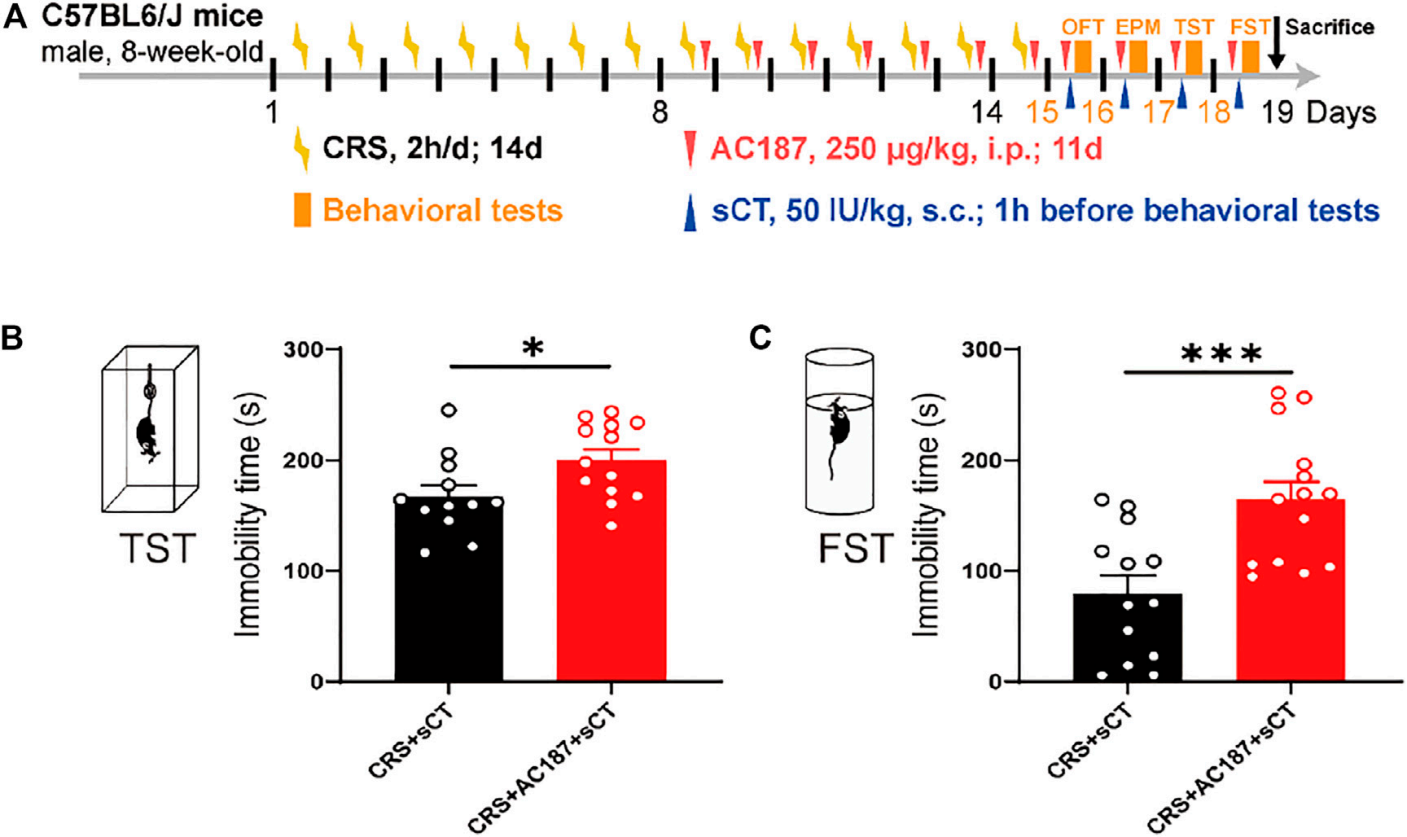
sCT exerted an antidepressant effect in CRS mice by activating AMYR. Timeline of CRS exposure, sCT, AC187 administration, and behavioral tests **(A)**. In TST, when AMYR was inhibited by AC187, the antidepressant effect of sCT was blocked (CRS + sCT: *n* = 12; CRS + sCT + AC187: *n* = 13) **(B)**. In FST, the antidepressant effect of sCT was blocked when the AMYR inhibitor AC187 was administered (CRS + sCT: *n* = 13; CRS + sCT + AC187: *n* = 14) **(C)**. The data were analyzed by unpaired *t*-test. *p < 0.05, ***p < 0.001. sCT, salmon calcitonin; CRS, chronic restraint stress; AMYR, amylin receptor; TST, tail suspension test; FST, forced swimming test.

**FIGURE 8:**
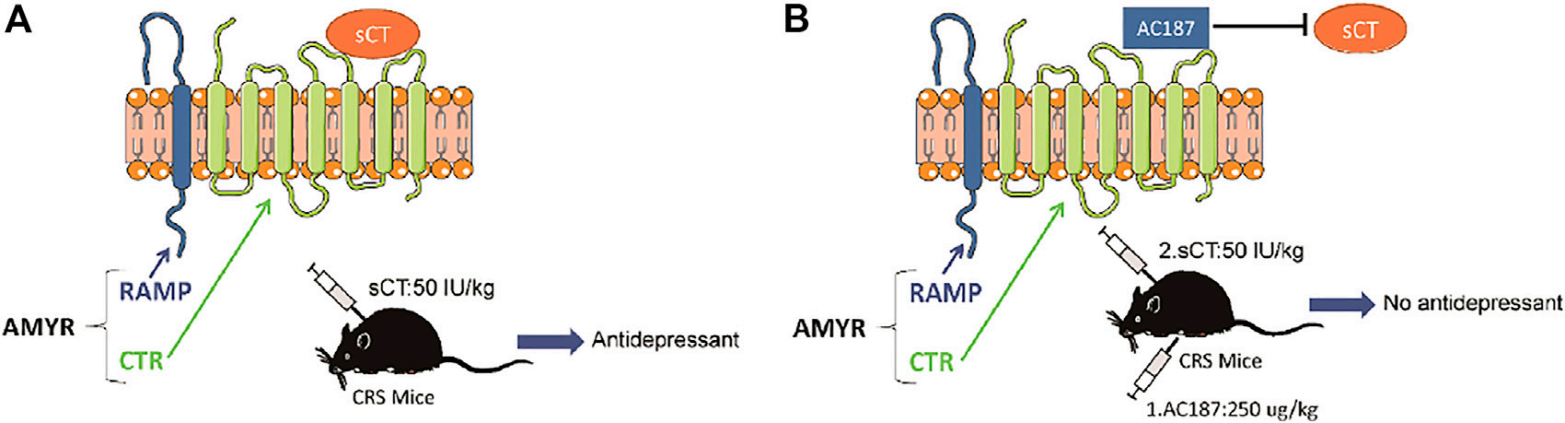
Working model of the sCT antidepressant effect by activating amylin receptors in CRS mice. sCT can activate AMYRs to play an antidepressant role **(A)**. AC187 eliminates the antidepressant effect of sCT **(B)**. sCT, salmon calcitonin; CRS, chronic restraint stress; AMYRs, amylin receptors.

AMYRs can be activated by their agonist peptides, such as sCT, or suppressed by the antagonists including AC187, AC66, and CGRP. Among them, the AC187 has the most antagonistic effect (Hay et al., 2005). As was observed in Figure 5, sCT could decrease the immobility time of the depressive-like mice. When AMYRs were inhibited by AC187, such an antidepressant effect of sCT was potently stymied [t_(23)_ = 2.353, *p* < 0.05, Figures 7A,B]. Similarly, in the FST, when AMYRs were inhibited by AC187, the antidepressant effect of sCT was significantly blocked [t_(25)_ = 3.744, *p* < 0.001, Figure 7C]. Collectively, these results delineated that sCT exerted antidepressant effects *via* AMYRs (Figures 8A,B).

## Discussion

Depressive disorders are a complex of neuropsychiatric diseases that affect a large population, while the current pharmacotherapies have a major limitation, a long working latency. In the present study, to evaluate the antidepressant potential of acute sCT injection, we first reproduced an animal model of CRS (Figure 1) with no locomotion and anxiety phenotypes (Figure 2) but with a significantly declined level of endogenous calcitonin in the serum and cerebral cortex (Figure 3). Further, we showed that the sCT treatment did not alter locomotor activity and anxiety in the non-stressed mice (Figure 4), nor in the CRS group (Supplementary Figure S2). Interestingly enough, we found that sCT did reduce the immobility time of the CRS mice during FST and TST analyses (Figure 5), demonstrating its antidepressant potential. Although no changes of CTR expression have been observed in various depression-associated brain regions in the stressed mice (Figure 6), the AMYR antagonist, AC187, potently reversed the antidepressant effect of sCT, suggesting the AMYR-function-dependent role of sCT (Figure 7). Of note, our results showed that AC187 did not affect the immobility time of CRS mice in TST and FST, suggesting that AC187 alone did not induce depressive-like behavior changes in CRS mice (Supplementary Figure S3G, H). Furthermore, the locomotion and anxiety levels in CRS mice were not affected by AC187 (Supplementary Figure S3B, C, E, F). Similarly, AC187 did not affect locomotion, anxiety level, and depressive-like behavior in WT mice (Supplementary Figure S4).

Calcitonin is a single peptide hormone secreted by parafollicular or C cells of the thyroid gland and composed of 32 amino acids (Srinivasan et al., 2020). sCT derived from salmon has a greater affinity for AMYRs than calcitonin from other species and has longer half-life elimination, which is widely used in the clinical treatment of bone diseases (Andreotti et al., 2006). To this end, this study investigated the potential antidepressant effects of sCT, known as a hormone for the regulation of serum calcium concentration. Furthermore, it has been reported that calcium level in serum is increased in people with depression (Joffe et al., 1996). This may be due to deficiencies in calcium’s regulatory ability in depressed patients. Our study found that calcitonin level was decreased in both the serum and the cerebral cortex after CRS (Figure 3). Similar to the results of our study, in depressed patients, the calcitonin level is decreased in the cerebrospinal fluid (Mathe et al., 2002). Therefore, it is reasonable to postulate that dysregulation of the calcitonin level may lead to hypercalcemia in depressed patients.

Calcitonin has a profound effect on the physiological function of the central nervous system. For instance, sCT can affect food intake through its effects on the NAc, VTA, and lateral dorsal tegmental area (Mietlicki-Baase et al., 2013; Mietlicki-Baase et al., 2015; Reiner et al., 2017). Moreover, sCT regulates alcohol intake by activating the mesolimbic dopamine system (Kalafateli et al., 2019a; Kalafateli et al., 2021). CGRP, as a member of the calcitonin family, has significant antidepressant effects in the depression model (Hashikawa-Hobara et al., 2015). Calcitonin and the CGRP are amino acid peptides encoded by the same gene and share a similar structure. Calcitonin mRNA is expressed in thyroid C cells, but CGRP mRNA is mainly produced in neurons, while they can bind to the same type of receptors (Coleman et al., 2003). These data support our hypothesis on calcitonin/CGRP against stress-induced depressive-like behaviors. Indeed, we found that acute sCT administration alleviated depressive-like phenotypes in CRS mice (Figure 5). Particularly remarkable, sCT did not affect the locomotion and anxiety level in the stressed and non-stressed mice (Figure 4). In keeping with our observation, *Kalafateli* et al. found that sCT treatment for 5 days did not affect the locomotion of WT mice (Kalafateli et al., 2020). In addition, injections of sCT to activate the VTA AMYRs did not reduce the time of animals spent in the inner zone or the number of entries to the inner zone (Mietlicki-Baase et al., 2017). Thus, it is thought that sCT or its receptor is not involved in the anxiety-related neural circuits.

The CTR family is extremely complex, including CTR and CTR-like receptors and three RAMPs (Garelja et al., 2021). AMYR consists of a core CTR and one out of three RAMPs and is expressed in the whole brain including reward-related regions (Kalafateli et al., 2021). Similarly, we found that CTR was universally expressed in the brain including PFC, cerebral cortex (without PFC), and hippocampus, but the levels of its expression were not changed in the CRS mice (Figure 6), suggesting that the depressive disorder is not significantly related to or dependent on the expression of CTR in the brain of CRS mice. In addition, agonists mainly work through enhancing the intrinsic activity *via* changing the conformation of the protein molecule, instead of modulating the expression level of the receptor, if it is applied in a relatively short period and limited time. Up until now, sCT is one of the most potent AMYR agonists, and compared to calcitonin in humans and rats, sCT (i.e., from salmon) activates the AMYR more effectively and consistently (Andreassen et al., 2014; Gydesen et al., 2017). Nowadays, small-molecule peptide ligands have been widely studied in depression, owing to the ability to cross the blood–brain barrier and specifically bind to their receptors (Holmes et al., 2003; Nwokafor et al., 2020). They could be therapeutic candidates for the treatment of depression. In this study, we delineated the potential role of the calcitonin–AMYR axis in depression by exploiting both the agonist (e.g., sCT) and the inhibitor/antagonist of AMYR (e.g., AC187) (Hay et al., 2005). We have demonstrated that the AC187 abolished the antidepressant effects of sCT (Figure 7), indicating that AMYRs play a functional role in the treatment of depression, although the expression level of AMYRs remained unchanged in major brain regions.

In summary, our study reveals a new role of sCT in the antidepressant effect by interacting with its receptors AMYRs. Our results may pave the way for future studies on determining the downstream mechanism of the AMYR, as well as for clinical research into potential targeted therapeutic strategies for the treatment of depression.

## Conclusion

The present study demonstrated that sCT rescued the depressive-like behaviors in the CRS mouse model of depression; however, the AC187, a potent antagonist of AMYRs, significantly eliminated the antidepressant effects of sCT. Therefore, our results indicated that sCT may exert rapid antidepressant effects by activating AMYRs.

## Data Availability

The original contributions presented in the study are included in the article/Supplementary Material, further inquiries can be directed to the corresponding authors.
