# Supplementary material for: Salmon Calcitonin Exerts an Antidepressant Effect by Activating Amylin Receptors
Source: Front Pharmacol. 2022 Feb 14;13:826055. doi: 10.3389/fphar.2022.826055 (PMC8883047; doi:10.3389/fphar.2022.826055)
Supplement: Supplementary file 1 [file DataSheet1.zip › Supplementary Material-The latest/Laboratory Animal Ethics Review Resolution of SUSTech.PDF]

南方科技大学实验动物伦理审查决议书

Laboratory Animal Ethics Review Resolution of SUSTech

决议编号<br>Resolution number	SUSTC-JY2019097		
实验动物<br>Laboratory animal	小鼠		
课题名称<br>Program name	降钙素的抗抑郁作用		
动物实验项目名称<br>Animal protocol name	降钙素抗抑郁作用的行为学测试以及 OCN 的表达水平检测		
申请人<br>Applicant	张冬		
受理编号<br>Acceptance number	SUSTC-2019-075		
申请时间<br>Application time	2019 年 3 月 19 日		
是否通过初审<br>Whether to pass the preliminary review	☑通过 **Agree**<br>☐不通过 **Disagree**		
会议或通讯审查的时间<br>Time of meeting or newsletter review	2019 年 4 月 16 日		
审查决议<br>Review resolution	☑可以进行实验 **Approved**<br>☐调整方案后，可以进行实验 **To be revised**<br>☐ 不同意 **Disapproved**		
主任或授权的副主任签名<br>Signature of the Chairman or authorized deputy Chairman	[signature]	日期<br>Date	2019年 4月 16日
