# Supplementary material for: Salmon Calcitonin Exerts an Antidepressant Effect by Activating Amylin Receptors
Source: Front Pharmacol. 2022 Feb 14;13:826055. doi: 10.3389/fphar.2022.826055 (PMC8883047; doi:10.3389/fphar.2022.826055)
Supplement: Supplementary file 2 [file DataSheet2.ZIP › Supplementary Material-FIGURE S1-S4.docx]

**Salmon Calcitonin** **Exerts an Antidepressant Effect by** **Activating Amylin Receptors**

Supplementary Material-FIGURE S1-S4

**
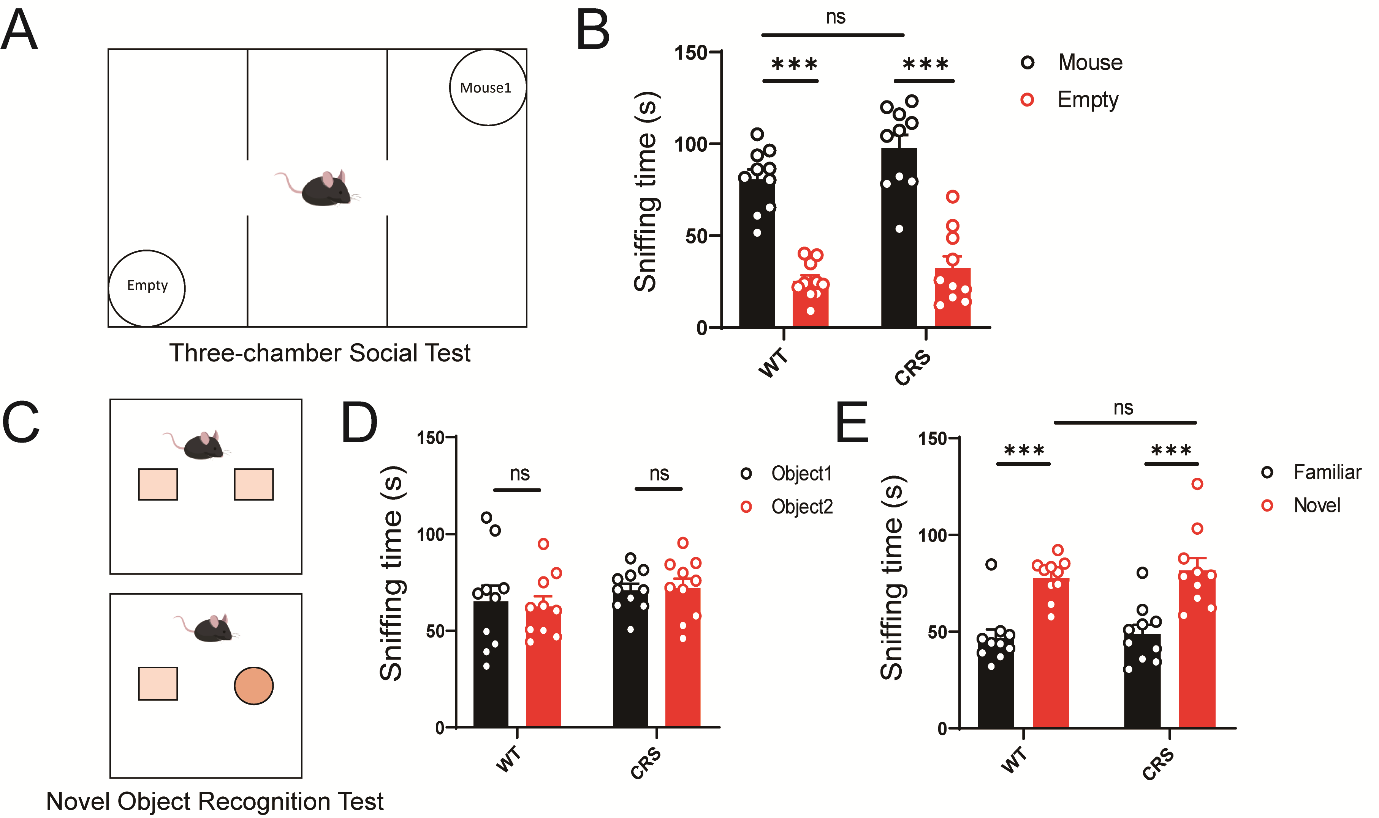
**

**FIGURE S1 There were no significant deficits in social ability and cognitive memory in mice with a depressive-like phenotype.** In the three-chamber social interaction test, similar to normal control mice, the CRS mice spend more time on interacting with a con-specific mouse in the second phase of the three-chamber social test (WT: n=10; CRS: n=10) (A-B). In the first phase of NOR test, there were no significant differences in exploration of the two identical objects in both CRS and control groups (WT: n=10; CRS: n=10) (C-D). In the second phase of novel object recognition (NOR) test, both CRS and control mice showed a preference to the new object, showing there were no significant deficits in cognitive memory of CRS mice (E). The data were analyzed by unpaired *t* test. **** p*<0.001.


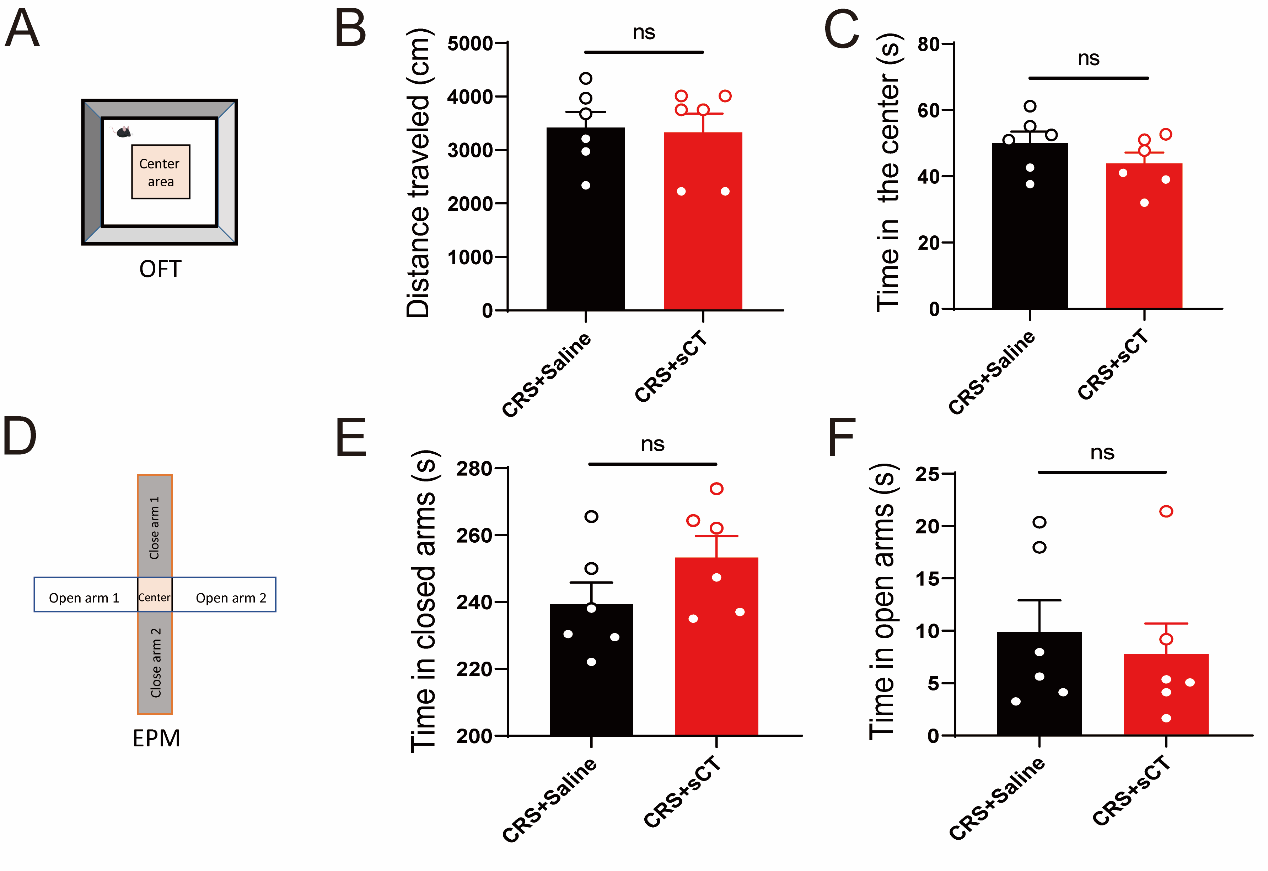


**FIGURE S2 sCT did not affect the locomotion and anxiety level in CRS mice.** Scheme of the OFT (A). The distance travelled in OFT did not change with application of sCT (CRS + Saline: n=6; CRS + sCT: n=6) (B). The time spent in the center area not significant change after the administration of sCT in CRS mice (C). Scheme of the EPM (D). Time in closed arms did not change after the administration of sCT (CRS + Saline: n=6; CRS + sCT: n=6) (E). sCT did not affect the time spent in the open arms of CRS mice (CRS + Saline: n=6; CRS + sCT: n=6) (F). The data were analyzed by unpaired *t* test.


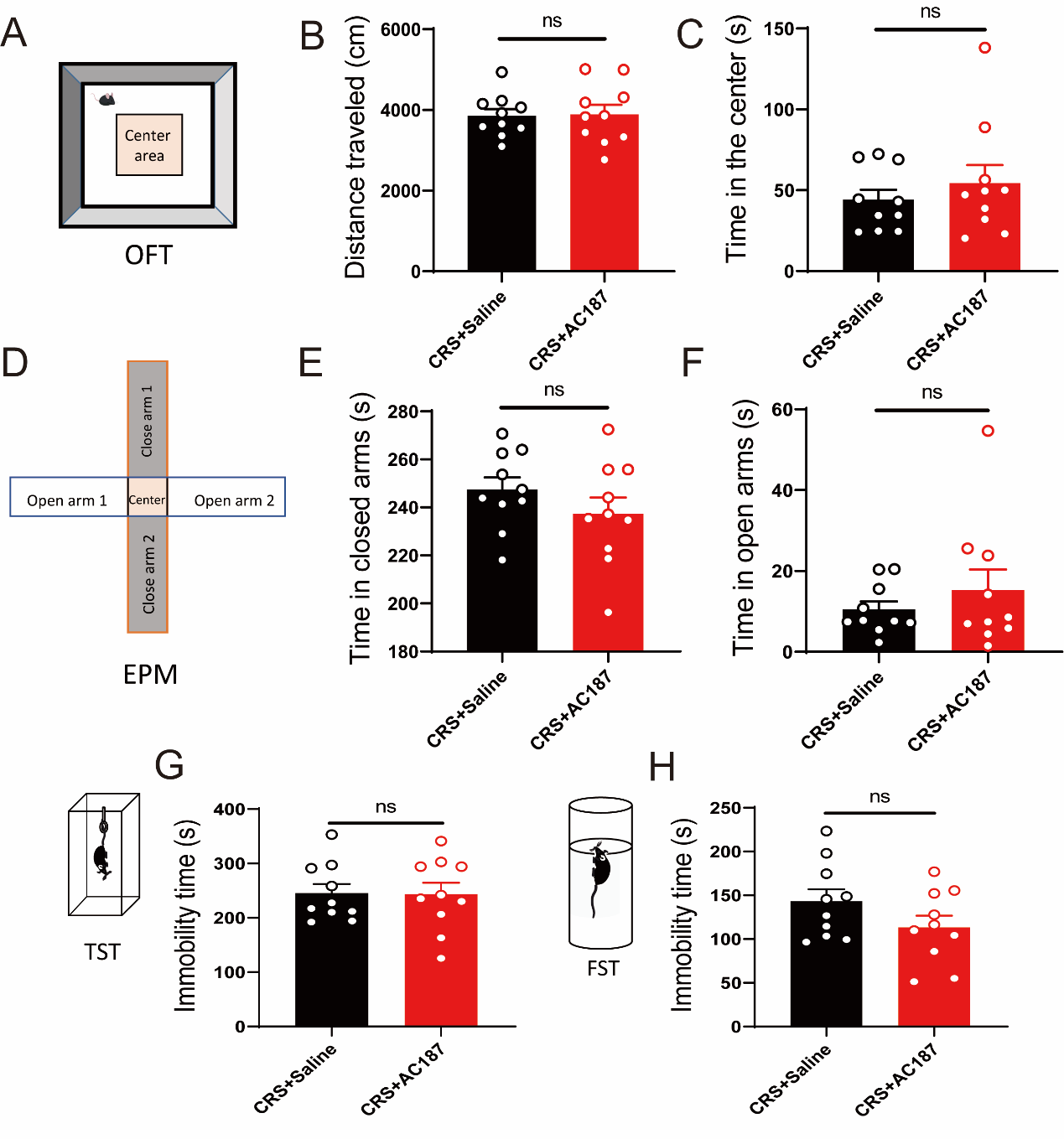


**FIGURE S3 AC187 did not affect the locomotion, anxiety level and depressive-like behaviors in CRS mice.** Scheme of the OFT (A). In OFT, the distance traveled of CRS mice did not significant change with application of AC187 (CRS + Saline: n=10; CRS+AC187: n=10) (B). Time in the center did not change after the administration of AC187 in OFT (CRS + Saline: n=10; CRS+AC187: n=10) (C). Scheme of the EPM (D). Time in closed arms did not change after the administration of AC187 in CRS mice (CRS + Saline: n=10; CRS+AC187: n=10) (E). AC187 did not affect the time spent in the open arms of CRS mice (CRS + Saline: n=10; CRS + AC187: n=10) (F). In TST, AC187 did not affect the immobility time of CRS mice (CRS + Saline: n=10; CRS+AC187: n=10) (A). In FST, the immobility time of CRS mice did not change with application of AC187 (CRS + Saline: n=10; CRS + AC187: n=10) (B). The data were analyzed by unpaired *t* test.

**
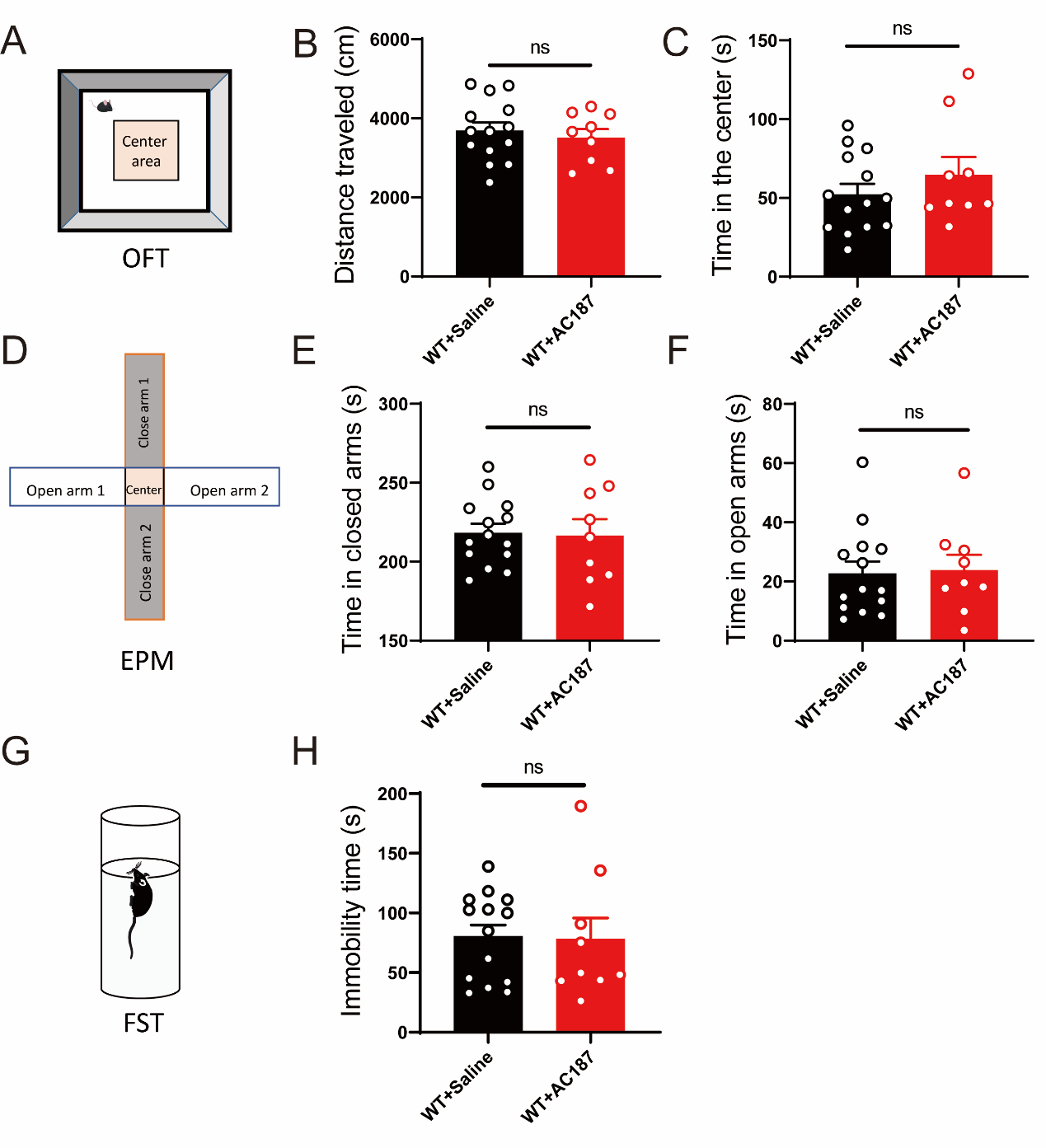
**

**FIGURE S4 AC187 did not affect the locomotion and anxiety level and nor induce depressive-like behavior in WT mice.** Scheme of the OFT (A). The distance travelled in OFT did not significant change with application of AC187 in WT mice (WT + Saline: n=14; WT + AC187: n=9) (B). Time in the center did not change after the administration of AC187 (C). Scheme of the EPM (D). AC187 did not affect the time spent in the closed arms of WT mice (WT + Saline: n=14; WT + AC187: n=9) (E). Time in open arms did not change with application of AC187 (WT + Saline: n=14; WT + AC187: n=9) (F). Scheme of the FST (G). In FST, AC187 did not induce depression-like behavior in WT mice (WT + Saline: n=14; WT + AC187: n=9) (H). The data were analyzed by unpaired *t* test.
